# Chemometric analysis of cow dung ash as an adsorbent for purifying biodiesel from waste cooking oil

**DOI:** 10.1038/s41598-017-09881-z

**Published:** 2017-08-25

**Authors:** A. Avinash, A. Murugesan

**Affiliations:** 1 Department of Mechanical Engineering, KPR Institute of Engineering and Technology, Arasur, Coimbatore, 641 407 Tamil Nadu, India; 2 Department of Mechanical Engineering, K.S.Rangasamy College of Technology, Tiruchengode, 637 215 Tamil Nadu, India

## Abstract

Taraditionally, the water-soluble contaminants of biodiesel are treated by water wash method. However, water wash method ends up in an aqueous effluent, which might then cause a harmful environmental impact. As a consequence, waterless purification of biodiesel has triggered primary interest in biodiesel manufacturing process. To address this issue, an endeavour has been made in this work to investigate the waterless purification of biodiesel from waste cooking oil using cow dung ash at different concentration of 1, 2, 3 and 4 wt/wt %. The optimum concentration of cow dung ash for biodiesel purification was found through chemometric analysis by comparing the Fourier transform infrared transmission (FTIR) spectral characteristics of cow dung ash with the water treated FTIR. It was observed from the experimental study that 1 wt/wt % of cow dung ash exhibited similar structural characteristics as that of traditional water treated method of biodiesel purification. Therefore, bio-waste cow dung ash is an effective adsorbent in purifying biodiesel analogous to traditional water washing technology.

## Introduction

In the past few decades, the rapid depletion of easily accessible underground carbon reserves and detrimental effects on environmental pollution impose the use of alternative renewable energy sources^1, 2^. In this perspective, biodiesel is one among the alternative renewable energy sources which have been widely used in many countries of the world^3^.

Biodiesel is a renewable and biodegradable fuel for use in existing automobile and stationary engines^4^. It consists of mono-alkyl (methyl or ethyl) esters of long-chain fatty acids typically produced by chemically reacting lipids (e.g., vegetable oil or animal fat) with an alcohol (methanol or ethanol)^5, 6^. Biodiesel is most commonly produced by transesterification of vegetable oil or animal fat feedstock through the different methods such as common batch process^7^, supercritical process^8^, ultrasonic irradiation^9^ and microwave methods^10, 11^. Among these methods, transesterification by the batch process was noted as the most economic and simple method to overcome problems due to direct use of oil in diesel engines^12^. Transesterification is the process of displacement of glycerol with simpler alcohol in the presence of a catalyst to chemically break the molecule of fat into esters with glycerol as a by-product^13^.

After glycerol removal by the separation method, the trace impurities such as unreacted alcohol, unreacted glycerides and unreacted catalyst present in biodiesel are traditionally removed by water washing. Several studies have reported that multiple water washes are required to achieve satisfactory impurity removal from unpurified biodiesel. Some cases may require as many as six individual washes^14^. Consequently, water washing method produces a large quantity of biodiesel treated wastewater that requires treatment before reuse and causes the operational problem (emulsion)^14^. Recently, several alternative “waterless” purification methods have been developed, such as ion exchange resins and adsorbent treatment^15^.

In the adsorbent method of biodiesel purification, commercial magnesium silicate (Magnesol) and silica were widely used^16, 17^. These adsorbents primarily consist of basic and acidic adsorption sites which can easily attract polar substances such as glycerol and unreacted methanol^17^. Kucek *et al*.^18^ achieved a significant reduction in monoglyceride and bond glycerin by adding 2 wt/wt % of Magnesol in unpurified biodiesel by stirring the mixture continuously for 20 min at 65 °C and then the Magnesol was filtered from biodiesel. Predojevic *et al*.^19^ assessed the use of silica gel to purify biodiesel and compared the results of silica gel purification to that of water washing method. In their study, it was found that there was a significant change in the acid value of biodiesel after purification by silica gel. However, there was no significant change in density, kinematic viscosity, iodine number, water content, saponification number due to purification by silica gel. Recent investigations by researchers portray that the use of silica- based agricultural waste products have shown success in the removal of impurities from biodiesel as reported by Márcia *et al*.^20^. In their work, the researchers have used rice husk ash as an adsorbent to purify waste frying oil biodiesel.

On the whole, the adsorption process is one of the efficient methods to remove the water-soluble contaminants present in biodiesel. However, there is a need to carry out further studies on biodiesel purification by adsorption using low-cost and eco-friendly adsorbents. In this regard, cow dung has several imperative properties which have been in use since ancient times. It is used as manure for agricultural purpose and in the production of biogas. It is used to repel mosquitoes and as a low-cost thermal insulator. Cow dung is also a possible constituent in the manufacture of adobe mud brick housing. In addition, cow dung ash (CDA) is used as an adsorbent for sequestering heavy metals present in wastewater^21^. Thus, the advantage of utilising cow dung ash as an adsorbent is not only revolving around its low economic value but also can stop the environmental issue of foul odour ensuing from it.

Based on these considerations, the main objective of present work is to purify biodiesel produced from waste cooking oil using CDA as an adsorbent at different concentration of 1, 2, 3 and 4 wt/wt % and compare their FTIR structural characteristics with water treatment method. In this work, CDA was prepared by burning cow dung cakes in the muffle furnace (500^o^ C for 2 h) and adsorption studies were performed by the batch technique using CDA as an adsorbent. Also in this work qualitative analysis (principle component analysis and hierarchical cluster analysis) and quantitative analysis (partial least square regression) were done through chemometric analysis of FTIR spectral data to find the optimum concentration of cow dung ash which demonstrates similar structural characteristics as that of traditional water purification method.

## Results and Discussion

### X-ray powder diffraction (XRD)

**Figure 1:**
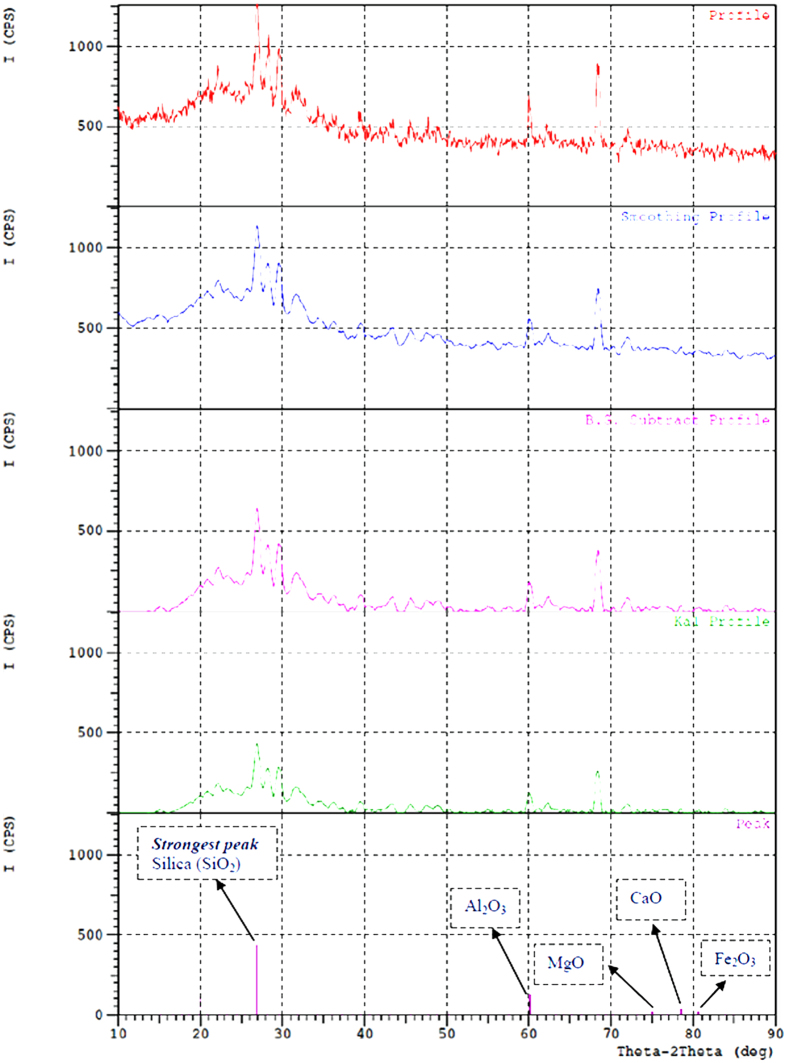
XRD pattern of cow dung ash.

The XRD analysis of the CDA was obtained with Copper (Cu) target at 40 kV, 30 mA under continuous scan mode with a scan range of 10–90 degree, and a scan speed of 10 degree/minute. The XRD pattern of CDA is shown in Fig. 1. This 2θ versus Intensity (I) (CPS - Counts per second) plot shows the profiles and peaks of compounds identified in XRD. Also, experimental XRD peaks of CDA at 500^o^ C were indexed with Joint Committee on Powder Diffraction Standards (JCPDS) file. Table 1 shows the comparison of planar spacing (d-spacing) and strongest peak intensities of CDA with standard compounds. The planar spacing values were calculated from Bragg’s law of diffraction (nλ = 2d sinθ). It can be seen from Table 1 that silica (SiO_2_) is a major phase in CDA followed by Al_2_O_3_, MgO, CaO and Fe_2_O_3_.

**Table 1:** Comparison of d-spacing, position and strongest peak intensities of CDA with standard compounds.

Compound	JCPDS	hkl	d-spacing (nm)		Position (2θ)		Relative intensity (%)	
			Standard	Experimental	Standard	Experimental	Standard	Experimental
SiO_2_	(46–1045)	101	0.33434	0.331583	26.639	26.8662	100	100
Al_2_O_3_	(10–0173)	122	0.15140	0.15147	61.164	61.10	5	6
MgO	(45–0946)	311	0.12698	0.126521	74.689	75.01	5	5
CaO	(37–1497)	400	0.12025	0.121854	79.662	78.4178	6	7
Fe_2_O_3_	(33–0664)	128	0.11896	0.119219	80.709	80.5	5	4

### Energy-dispersive X-ray spectroscopy (EDX)

**Figure 2:**
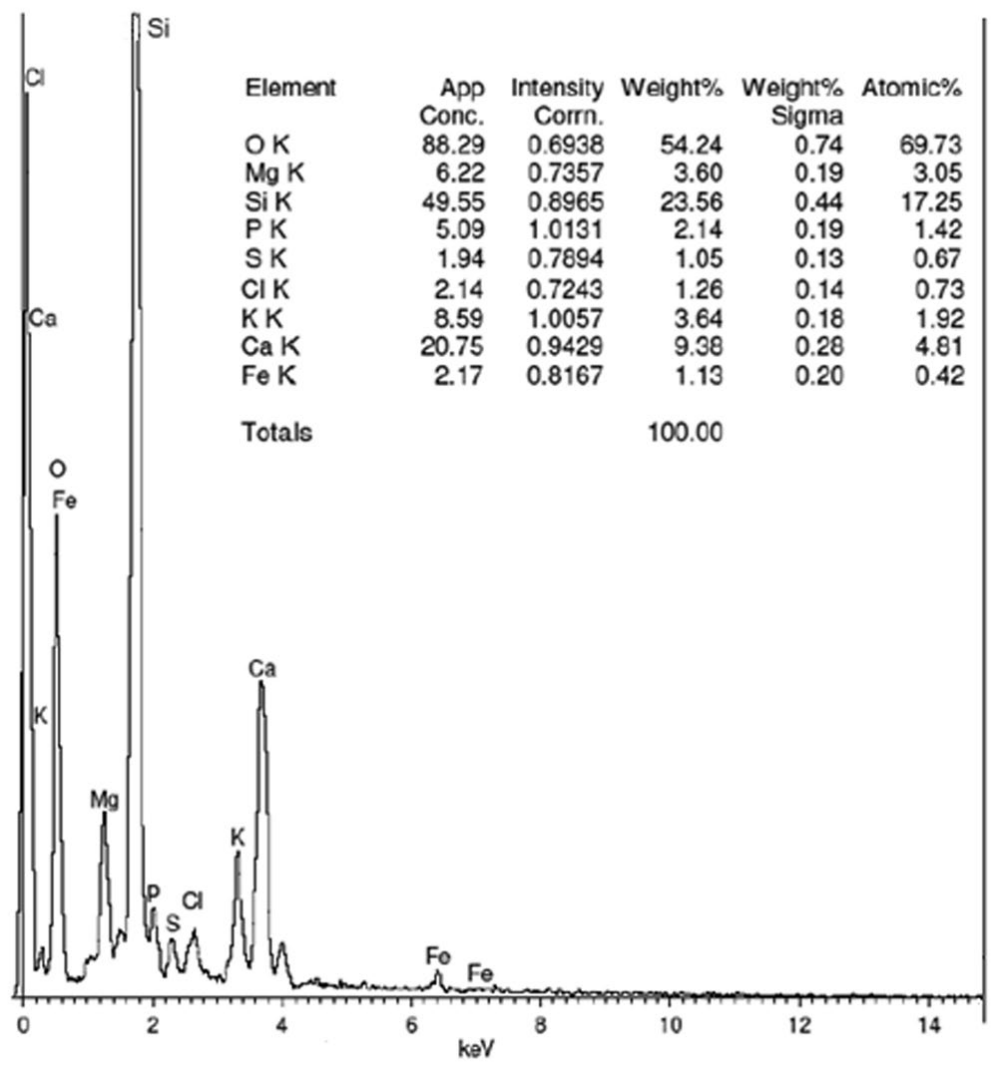
EDX spectrum of cow dung ash.

The percentage composition of various elements present in CDA was characterised by EDX. The EDX spectrum of CDA (Fig. 2) shows various elemental peaks with the major Si peak (Kα = 1.74 keV) and O peak (Kα = 0.525 keV). The percentage composition of various elements present in CDA is also shown in Fig. 2. The percentage of Si in the ash is higher with some metallic impurities in minor amounts.

### FTIR characterisation of biodiesel treated wastewater

**Figure 3:**
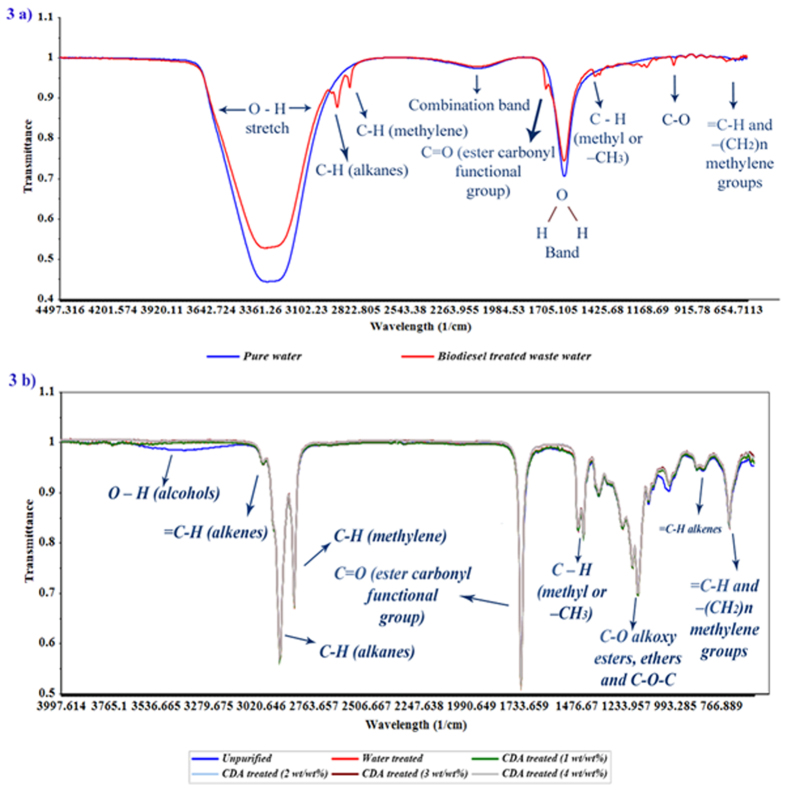
(**a**) FTIR spectra of pure water and biodiesel treated wastewater (**b**) FTIR spectra of biodiesel samples.

In this work, pure distilled water before and after biodiesel treatment is characterised by FTIR to identify the transport of organic and inorganic impurities to pure distilled water after biodiesel purification. Figure 3(a) shows the FTIR spectrum of pure distilled water and the spectrum of biodiesel treated wastewater. Also, the functional groups present in pure and biodiesel treated wastewater are marked in Fig. 3(a). Pure distilled water showed a strong IR absorbance band at 3319.48 cm^−1^(O-H stretching) and around 1636.63 cm^−1^ (H-O-H bending). Also, pure water exhibited combination band centred at 2117.78 cm^−1.^ On the other hand, the strong band for biodiesel treated wastewater shifted to a higher wavelength, around 3331.57 cm^−1^(O-H stretching) and around 1638.44 (H-O-H bending). Also, the combination band of biodiesel treated wastewater shifted to 2120.26 cm^−1^. This peak shift denotes the dilution and electron transfer effect of methyl ester in biodiesel to pure distilled water. This analysis discloses that there is a need to go for waterless purification of biodiesel to overcome the aqueous effluents released by water wash technique.

### FTIR characterisation of water and CDA treated biodiesel samples

The mid-infrared spectral data (4000–400 cm^−1^) have been used to identify functional groups and the bands corresponding to vibration in the unpurified, water purified and ash treated biodiesel samples. Figure 3(b) shows peaks identified from the spectra of 6 biodiesel samples. The typical peak at 3397 cm^−1^ with stretching mode of vibration seen in the unpurified spectrum is attributed to the presence of alcohol (O-H) group. This peak is absent for the water purified and ash purified samples. This clearly indicates that the ash treatment of biodiesel removes the unreacted/impure alcohol present in the unpurified biodiesel similar to water treatment process. The removal of impurities present in unpurified biodiesel was achieved by ash treatment because of the presence of high percentage of silica in cow dung ash. In this work, it was observed that FTIR spectrum of water treated and ash treated samples are structurally similar and it is very difficult to visually discriminate the FTIR spectral data of the samples (Fig. 3(b)). Thus, the chemometric analysis was carried out with the spectral data to classify the samples and to find the optimum weight percentage of cow dung ash to be used for biodiesel purification which exhibits similar structural characteristics as that of water purified biodiesel.

### Characterisation of biodiesel

In this work, the methyl ester content of biodiesel was noted as 95.05 ± 0.26%. Indeed, the methyl ester content is not a very important parameter for determining the stage of purification because the purification process is no way associated with transesterification reaction and it does not have an effect on the methyl esters already formed. Thus, the biodiesel samples were characterised by acid value, saponification value, methanol content, water content, free and total glycerin. The biodiesel characterisation results of unpurified, water purified and ash treated biodiesel samples are presented in Table 2. It can be observed from Table 2 that 1 wt/wt % CDA on biodiesel exhibited results analogous to that of traditional water washing technology.

**Table 2:** Analysis of biodiesel samples.

Purification method	Acid number (mg KOH/g)	Saponification value (mg KOH/g)	Methanol (%)	Water (mg/kg)	Free glycerin (%)	Total glycerin (%)
Unpurified	0.30 ± 0.01	212	0.74 ± 0.02	2247.4	0.00782 ± 0.00001	0.59 ± 0.02
Water purified	0.21 ± 0.01	204	0.015 ± 0.01	5365.2	0.00035 ± 0.00001	0.49 ± 0.01
CDA treated 1 wt/wt %	0.22 ± 0.01	205	<0.01	1879.5	0.00393 ± 0.00002	0.50 ± 0.01
CDA treated 2 wt/wt %	0.27 ± 0.01	206	<0.01	1881.2	0.00451 ± 0.00001	0.48 ± 0.01
CDA treated 3 wt/wt %	0.27 ± 0.01	207	<0.01	1880.8	0.00412 ± 0.00001	0.47 ± 0.01
CDA treated 4 wt/wt %	0.29 ± 0.01	207	<0.01	1881.6	0.00442 ± 0.00001	0.48 ± 0.01

### Chemometric analysis of spectral data

**Figure 4:**
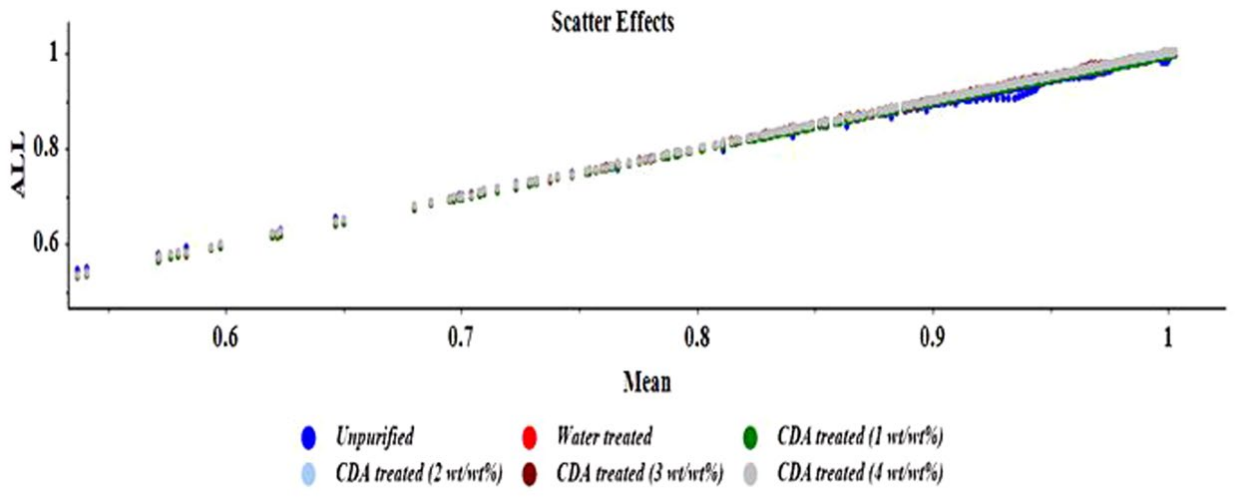
Scatter plot of biodiesel samples.

The chemometrics was constructed using preprocessing method*- Savitzky-Golay* (*SG*) *filtering with second polynomial order and 11 smoothing points*. Among different preprocessing methods, SG filtering was chosen because spectra in the dataset of all samples appear to have the similar scatter level as shown in Fig. 4 and all spectra were recorded on the same day and on the same lab so there were no overlapping bands in the raw spectra.

### Qualitative analysis

Before establishing quantitative model using partial least square regression (PLSR), unsupervised methods like principal component analysis (PCA) and hierarchical cluster analysis (HCA) were used as classification tools for analysing FTIR spectra of unpurified, water treated and ash treated biodiesel samples.

**Figure 5:**
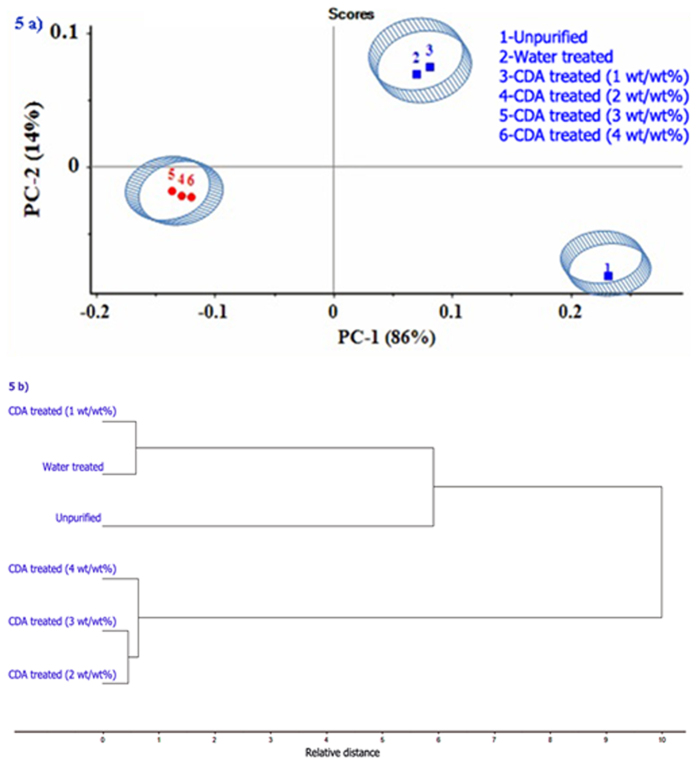
(**a**) Score plot of PCA (**b**) Dendrogram.

PCA is a well-known method of dimension reduction and data exploration^22^. To visualise the data trend of unpurified and purified biodiesel samples, a two-dimensional graph of samples using the first two principal components (PCs) was obtained, which is shown in Fig. 5(a). The PC1 and PC2 explained 86% and 14% of variables, respectively. This means that in PCA the first two principal components could possibly explain 100% of all sample information. Also from the score plot shown in Fig. 5(a) it can be seen that unpurified biodiesel is an outlier in the lower right quadrant followed by close clustering of water treated and ash treated (1wt/wt %) samples in the upper right quadrant. On the other hand, ash treated biodiesel samples (2, 3, 4 wt/wt %) formed another cluster in the lower left quadrant of the score plot. Thus, PCA discriminates structurally similar unpurified and purified biodiesel samples from each other using the score plot visually.

HCA was performed using the Euclidean distance as the distance measure and complete linkage strategy to link clusters within the dataset. Figure 5(b) shows the dendrogram of the spectral data. It can be seen from the dendrogram that the water purified and ash treated (1 wt/wt%) biodiesel formed one cluster and the ash treated (2, 3 and 4 wt/wt%) biodiesel samples formed another cluster. Overall, it is clearly seen from the PCA and HCA that the water purified and 1wt/wt% ash purified biodiesel sample exhibit similar structural characteristics.

### Quantitative analysis

**Figure 6:**
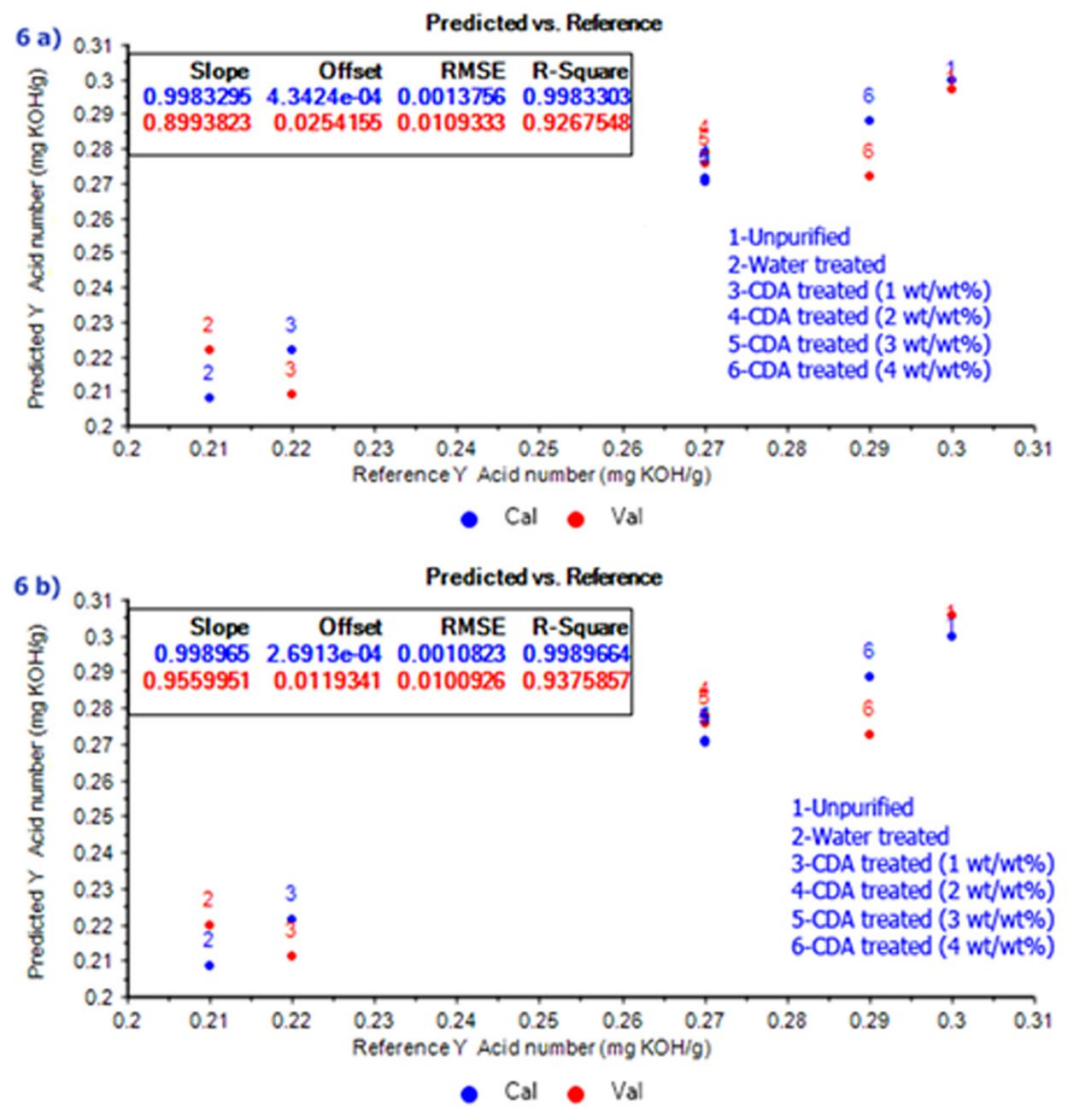
(**a**) PLSR model for raw data (**b**) PLSR model for preprocessed data.

Even though PCA and HCA explore the relationships between the spectral data, these methods cannot be used for the quantitative analysis. For this purpose, PLSR method is employed to develop a model. The PLSR model was developed for acid value alone because it was found from the present work and available literature that there was no significant change in water content, saponification number, methanol and glycerin content due to purification by silica-based adsorbents. However, there was a significant change in acid value^19, 20^. In this context, the models were generated for the acid value of biodiesel and compared using PLSR from raw and SG preprocessed data. These models could be used for the prediction of the acid value of biodiesel. Between the raw and SG preprocessed model, preprocessing had the best performance with a high correlation of prediction (RP = 0.937) and low root mean square error of prediction (RMSEP = 0.010) as shown in Fig. 6(a,b).

## Conclusion

Based on the present investigation on the use of cow dung ash as an adsorbent for purifying waste cooking oil, the following major conclusions are drawn:

- In this study cow dung ash was successfully used as an adsorbent for purifying biodiesel from waste cooking oil. The test results clearly indicated that cow dung ash in a concentration of 1 wt/wt % showed an excellent result for removal of impurities from biodiesel similar to that of water treatment. This result was possibly achieved in this work because of the high silica content of the CDA as confirmed by XRD and EDX analysis, which also accounts for its high adsorptive capacity.
- FTIR spectroscopy of biodiesel coupled with chemometrics was a helpful method to visually classify the highly correlated spectra and PLSR model developed from preprocessed spectra is considered as a useful tool for rapid detection of the acid value of biodiesel.
- At last, it is suggested from this work that biodiesel purification by cow dung ash has been shown to be environmentally friendly and a viable alternative substitute to water washing technology. For these reasons, it is important to explore and exploit other bio-waste silica-based adsorbents to purify crude biodiesel.

## Materials and Methods

### Cow dung ash preparation

Cow dung was collected from a grazing field near KPR farms, Coimbatore, Tamil Nadu state, India. Cow dung was air-dried properly and grounded to powder. The grounded cow dung powder (100 g) was taken in a silica cup and heated in the muffle furnace at 500 °C for 2 h. The resultant ash (CDA) was then allowed to cool to room temperature and stored in a desiccator to prevent it from absorbing moisture. In this work, the temperature of 500 °C is chosen because it was noted from the available literature that the temperature at 500 °C will improve the adsorption property of cow dung ash^23^. The CDA was characterised by XRD followed by EDX.

### Production of biodiesel

The transesterification process of waste cooking oil was done with a base catalyst (NaOH) and methanol. The reaction was carried out at the stirrer speed of 500 rpm, the reaction time of 60 min with 9:1 molar ratio and 0.75% NaOH (wt/wt of oil) as a catalyst at fixed reaction temperature of 65 °C^24^. The transesterified oil was transferred to a conical flask for the gravity separation of the biodiesel and glycerol. The unpurified biodiesel was then taken for the purification process.

### Water purification

A sample of 100 g of biodiesel was transferred to a separating funnel and washed thrice with pure distilled water. The washed biodiesel was heated at 90 °C to remove the water and stored for spectral analysis.

### Cow dung ash purification

The biodiesel purification using CDA was performed in a batch mode under heating at 65 °C and stirring for 20–30 min^17^ with different concentration of 1, 2, 3 and 4 wt/wt% of CDA. The concentration (wt/wt) percentage of CDA was calculated based on Equation (1). The purified biodiesel was filtered through a funnel with Whatman filter paper to remove the adsorbent and the samples were stored for the spectral analysis.

1 $${\boldsymbol{Concentration}}\,(\frac{{\boldsymbol{wt}}}{{\boldsymbol{wt}}}) \% =\,\frac{{\boldsymbol{mass}}\,{\boldsymbol{of}}\,{\boldsymbol{solute}}\,({\boldsymbol{CDA}})}{{\boldsymbol{mass}}\,{\boldsymbol{of}}\,{\boldsymbol{solution}}\,({\boldsymbol{biodiesel}})}\times {\bf{100}}$$

### Characterisation of biodiesel

Initially, the ester content of biodiesel was determined according to the method EN 14103. The acid value of biodiesel samples (unpurified and purified) was experimentally determined by titration procedure in accordance with EN 14104. In addition to acid value, saponification value (ASTM D 5558-95) of biodiesel samples was determined. The methanol content in biodiesel samples was determined according to method EN 14110 and water content by the Coulometric Karl Fischer method (EN 12937). Also, free glycerin and total glycerin in biodiesel samples were estimated by method EN 14105. All the procedures were done in duplicate and average values are presented.

### Chemometrics

The mid-infrared spectral data of the 6 biodiesel samples were used to perform the chemometrics using principal components analysis, hierarchical cluster analysis and partial least square regression using CAMO software (The Unscrambler X). The data from each sample contain 1667 variables; a total of 10002 variables were analysed. The analysis was based on a 6 × 1667 data matrix assembled so that each row corresponded to a sample and each column represented the spectral data at a given wavelength.
